# Expanding the Role Repertoire While Aging: A Drama Therapy Model

**DOI:** 10.3389/fpsyg.2021.635975

**Published:** 2021-03-05

**Authors:** Shoshi Keisari

**Affiliations:** ^1^Department of Gerontology, University of Haifa, Haifa, Israel; ^2^Department of Philosophy, Sociology, Education, and Applied Psychology (FISPPA), University of Padua, Padua, Italy

**Keywords:** drama therapy, life-review, older adults, playback theater, role theory, group intervention, narrative therapy, arts therapies

## Abstract

Drama therapy has been found to improve various facets of mental health while aging. It provides opportunities for personal growth and creative expression and enhances group relationships. Drama therapy is a widely acknowledged way to explore the life stories in late life. However, only a handful of studies have empirically explored the value of drama therapy for the aging population. This conceptual analysis was designed to address this need by developing a new integrative model of drama therapy. The analysis is based on the review of the results of four studies that explored the integration of life-review and playback theater as a drama therapy approach for older adults. The therapeutic process focused on the exploration of life-crossroads stories, a short unique technique which enables the participants to craft a harmonious view of their life stories in a short-term dramatic creative group process. Combining the four results yielded a multidimensional model which points to three potential transformative routes: the evolution of the life story, the evolution of improvised dramatic expression, and the expansion of social engagement. The transformative routes are described through the lens of *role theory* in drama therapy.

## Introduction

The extension of life expectancy has significantly increased the need to enhance the mental health of the aging population (World Health Organization, 2015, 2017). According to the tenets of positive psychology, one of the dominant schools of thought in gerontology (Araújo et al., 2017), the term *mental health* refers to both the hedonic and eudaimonic sides of well-being. Thus, mental health also refers to a positive development-while-aging process, and not only to the absence of mental illness, such as depressive symptoms and anxiety (Keyes, 2002, 2005). This type of attitude enables individuals to accept their lives as a whole and maintain personal growth, thus developing a positive identity despite the major challenges of this period of life (Erikson, 1959, 1982; Sneed and Whitbourne, 2003, 2005). The goal of the current work was to address this need by developing a new integrative model of drama therapy that is adapted for the aging population, to enhance mental health and to preserve positive identity. It is based on the synthesis of the results of four studies from a larger unified research project that explored the integration of life-review therapy and playback theater as a drama therapy approach.

### Integrating Drama Therapy and Life-Review in Old Age

Drama therapy is one of the creative arts therapies (CAT). It constitutes an active and experiential psychotherapy modality that involves the intentional and systematic use of drama/theater processes as a primary means of achieving psychological growth and change within a psychotherapeutic relationship (Emunah, 1994; Feniger-Schaal and Orkibi, 2019). In drama therapy sessions, individuals explore their life experiences in an in-depth manner through dramatic enactment, fantasy and imagination-based techniques to step into a character and tell a story from within this role (Pendzik, 2006; Landy, 2008). The literature indicates that drama therapy benefits a range of the aging population, from those who are high functioning to those who live with a physical, emotional, and cognitive challenges (Sandel and Johnson, 2014; Jaaniste et al., 2015; Keisari and Palgi, 2017; Dassa and Harel, 2019a, b; Dunphy et al., 2019). Drama therapy was also found to increase group involvement and positive relationships while aging (Mechaeil et al., 2009), and promote improved communication and a sense of confidence among those suffering from dementia (Lepp et al., 2003). Although the results of these studies point to the potential of drama therapy to enhance mental health, there are few evidence-based models that are adapted for the aging population (Dunphy et al., 2019).

The use of drama therapy to deepen life story work is an accepted practice. Narradrama, for example, combined the principles of narrative therapy, as developed by White and Epston (1990), and additional approaches from the art therapies (Dunne, 2000, 2003). Other works have emphasized the combination of narrative therapy, drama therapy (Milioni, 2001; Novy et al., 2005; Bird, 2010; Novy, 2018, 2019), and psychodrama (Brown-Shaw et al., 1999; Azoulay and Orkibi, 2015) as well as narrative therapy and playback theater (Barak, 2013). However, there is scant evidence-based literature on these combined approaches, specifically as related to the life stories of older adults.

The literature points to the central role of processing one’s life story as vital to the development-while-aging process, because it provides individuals with resources and promotes adaptation to changes throughout the life course (Butler, 1963; Erikson, 1982; De Medeiros, 2013). Accordingly, theatrical creative processes that bring the memories of older adults to life are used in a variety of settings, such as in “reminiscence theater” (Schweitzer, 2007), “autobiographical therapeutic theater,” (Harel, 2016) and “testimony theater” (Peleg et al., 2014). The aim of the current conceptual analysis was to provide an evidence-based model for drama therapy interventions, which would allow the participants to revive and explore their life-stories through a dramatic creative process in their own community.

The analysis is based on the review of four studies that implemented *Life-Review*, one of the leading approaches to therapeutic processes in late life (Butler, 1963; Erikson, 1982; Haber, 2006; Westerhof, 2015). Life review encourages the formulation of an integrative view of one’s life story by including life events from different periods in the life span and positive memories and achievements, alongside the acceptance of failures and harsh life events (Kenyon et al., 2010; Westerhof and Bohlmeijer, 2014). Therapeutic interventions based on life-review were found to be effective in improving mental health while aging (Korte et al., 2012; Zhang et al., 2015; Westerhof and Slatman, 2019). By creating a harmonious view of the past, present, and future, life achievements alongside failures and disappointments, the therapeutic work helps individuals to accept their life as a whole and enhance meaning in life, self-acceptance, positive identity and mental health (McAdams, 1993, 2001). In this context, *Role theory* is crucial to an understanding of the notions of mental health in drama therapy.

### Role Theory in the Context of Mental Health

One of the main goals in the drama therapy process is to expand the range of individuals’ role repertoire and to enable flexible movement between roles (Landy, 2009). The concept of *role* is central to drama therapy (Landy, 1993, 2009; Landy et al., 2003) and psychodrama (Moreno, 1961, 1985), as well as to theories deriving from sociology (Goffman, 1959) and psychology (Sarbin and Allen, 1968). According to role theory in drama therapy and psychodrama, a *role* is not just a social façade or creation but rather is the actual and tangible form of the *self*, comprised of a set of qualities representing the various facets of the individual (Moreno, 1961; Landy, 1993). Mental health, from the point of view of role theory in drama therapy, is characterized by an ability to express a variety of roles in daily life, and to live with ambivalence, contradictory tendencies, and paradoxes in the roles one expresses (Landy, 1993, 2009).

The need to face numerous changes in social roles while aging, challenges individuals’ mental health. The process in drama therapy strives to expand the range of roles individuals express and to enable flexible movement between them, making it possible for the individual to simultaneously accommodate a variety of roles (Landy, 1993, 2009). In other words, one can experience oneself as being old and, at the same time, feel young; one can comply with a role which requires dependency on others and at the same time take on a productive and independent role.

Similar to role theory in drama therapy, psychological approaches suggest that a *positive identity* integrates a wide range of different, sometimes conflicting, roles and relationships into a unified and purposeful whole (McAdams, 1993, 2001, 2019). This acceptance is highly significant when facing and coping with the major changes and losses that characterize aging. Thus, in order to strengthen positive identity and sense of continuity while aging, therapeutic interventions aim to enable participants to manifest multiple roles they may have embodied throughout their lifespan. At the same time, the therapeutic process should enable the exploration and experiencing of new roles, to enhance a sense of personal growth through the developmental process of aging. The potential transformations of the integrative process that combines life-review and drama therapy is discussed here through the lens of role theory in drama therapy.

## Overview of the Four Studies

This article presents a new integrative model that combines life-review with drama therapy. It is based on the results of four studies led by the author (Table 1), which explored the integration of life-review, life-stories and drama therapy for the aging population. The studies used one or two of the following guided frameworks: *playback theater* and *life-crossroads* stories.

**TABLE 1:** An overview of the four studies.

Authors, year	Study design	Participants	Setting	Inclusion criteria	Intervention	Control	Data collection and analysis	Main outcomes
Keisari and Palgi, 2017	Pre–post intervention	55 Participants with a mean age of 78.4, SD = 7.89 ranging from 62 to 93 years of age	Participants were members of three different settings: an adult day center, a social club, and a continuing care resident community	(a) Being capable of participating in verbal group; (b) achieving a score of 25 or more on the Mini Mental State Examination (MMSE > 24), indicating a normal cognitive level (Woodford and George, 2007); (c) participating in at least nine sessions, and without missing more than three sessions in a row	Life-review therapy based on the concept of *life-crossroads*, integrated with various drama therapy techniques including role playing, dramatic dialogs and monologs, playback theater, and psychodrama vignette of “empty chair” and “role reversal.” Twelve 90-min weekly sessions, co-moderated by a drama therapist and the setting’s social worker.	Care-as-usual treatment. No differences were found between the experimental and control groups before the therapeutic intervention on most of the variables. In relation to *self-acceptance* and *relationships with others*, the indices of the control group were more positive than those of the experimental group.	Self-report questionnaire assessing meaning in life, self-acceptance, relationships with others, depressive symptoms. Repeated measures ANOVA was used to examine the time differences for each measure as well as the differences between the experimental and the control groups. Analysis controlled for the demographic and setting differences.	Significant Time-Group interaction, validated the intervention’s effectiveness for all variables.
Keisari et al., 2018	A qualitative study based on the experiences of eight therapists	Eight therapists (drama therapists, a psychodramatist, a clinical psychology, a social worker and a group facilitator). Ranging from 2 to 23 years of practice	Groups were conducted in social clubs for the general older population; social clubs for Holocaust survivors, nursing homes for people with dementia, and adult day centers	(a) Therapists who conduct playback theater groups with older adults or use the techniques of playback theater in group therapy; (b) conducting a weekly group meeting for at least 6 months	Group therapy processes that were based on the playback theater ritual or that involves playback theater techniques to explore the life stories of older adults. Various durations of group processes and different session lengths		Semi-structured face-to-face interviews that focused on the therapeutic process and its main aspects from the therapist’s perspective. A grounded theory approach (Strauss and Corbin, 1990) was used for data collection and analysis.	The model yielded four major categories that represent the four key aspects of the process and its transformative effects: (1) **Reliving the life stories of older adults**, thereby generating therapeutic value from the testimonies and reconstruction of one’s life story in the dramatic improvisation; (2) **Playing within the stories of older adults** encourages fun, flexibility and self-development along with a sense of relevance and essentiality for older adults; (3**) Giving a stage to aging body** that results in a sort of awakening, and visibility through the creative process; (4) **A shared experience** is created for the older participants.
Keisari et al., 2020b	A randomized controlled design	78 Participants with a mean age of 79.60 years, SD = 6.89, ranging from 63 to 96	Participants were members of four adult day centers.	(a) a score of 25 or more on the Mini Mental State Examination (>24), indicating a normal cognitive level (Woodford and George, 2007); (b) an expressed interest in participating in a theater group and sharing their life stories with others; (c) usual visits at the setting on the intervention day; and (d) an absence of a clinical diagnosis of major psychiatric disorders and drug/alcohol abuse; (e) participating in at least nine sessions, without missing more than three sessions in a row.	An integrative intervention called *Life-Review Playback Theater*, which combines the key concepts of conducting playback theater with older adults with the principles of life-review therapy, using the concept of *life-crossroads*. Twelve 90-min weekly sessions, co-moderated by a drama therapist and the setting social worker	Care-as-usual treatment. No differences were found between the experimental and control groups before the therapeutic intervention, except for differences in well-being that was higher in the control group	Self-report questionnaire assessing meaning in life, self-acceptance, relationships with others, personal growth, positive and negative affect, depressive symptoms, and loneliness before, immediately after, and 3 months after the intervention A generalized estimating equations (GEE) framework was used to study the time differences of each measure as well as the differences between the experimental and the control groups. Analysis controlled for demographic and setting differences.	Significant time-group interaction validated the intervention’s effectiveness for improving positive mental health indices, as well as depressive symptoms. This improvement remained stable 3 months after the intervention. Negative affect and loneliness were more durable and did not change throughout the experiment.
Keisari et al., 2020a	An arts-based study that explored the participants’ experiences.	27 Participants with a mean age of 79.34 years, ranging from 63 to 96. Most had no previous experience taking part in theater-like groups. 13 adult-day-center staff members of the settings	Participants were members of three adult day centers	(a) A score of 25 or more on the Mini Mental State Examination (>24), indicating a normal cognitive level (Woodford and George, 2007); (b) an expressed interest in participating in a theater group and sharing their life stories with others; (c) usual visits at the setting on the intervention day; and (d) an absence of a clinical diagnosis of major psychiatric disorders and drug/alcohol abuse; (e) participating in at least eight sessions	An integrative intervention called *Life-review Playback Theater*, which combines the key concepts of conducting playback theater with older adults with the principles of life-review therapy, using the concept of *life-crossroads*. Twelve 90-min weekly sessions, co-moderated by a drama therapist and the setting social worker		Sessions were videotaped to capture the lived experience of the creative process and were analyzed with the participants in post-intervention interviews. Staff members were interviewed in three post-intervention focus groups to validate the out-of-group effects of the intervention. Data were analyzed using a thematic analysis method (Braun and Clarke, 2006).	Three main themes emerged that point to the potential transformations that occurred during the process: (a) **evolution of the life story** that reveals four sub-themes: (1) sharing and carrying of past experiences, (2) the embodied life-story, (3) resolving unfinished business, and (4) challenges in the process; (b) **evolution of playfulness** that reveals five sub-themes: (1) playing as a means of self-expression, (2) role playing as inducing the experience of having a role in life, (3) being in a role engages the aging body, (4) a shared expression, and (5) challenges in the process; (c) **expansion of social engagement** that reveals three sub-themes: (1) a fusion of experiences, (2) engagement with the adult day center community, and (3) engagement with the family.

### Playback Theater

Drama therapy is a broad therapeutic field, including a wide variety of dramatic tools and approaches to encourage personal growth and the development of coping resources (Bailey, 2007). The research process in studies 2, 3, and 4 dealt with on *playback theater* as a drama therapy method that provides a safe and creative space in which older adults can explore their personal experiences through their involvement in improvised dramatic action (Salas, 1993; Kowalsky et al., 2019). Playback theater is a form of improvisational theater in which a group of actors creates a theatrical improvisation in response to a personal story (Fox and Dauber, 1999). Over the years, playback theater has become a popular drama therapy group approach (Pendzik, 2008). The groups usually meet on a regular basis and in a fixed setting, and the sessions are conducted by a drama therapist. In each session, each member participates as a teller, a performer, or a spectator (Kowalsky et al., 2019). In addition, playback theater has a ritual framework that defines the roles of the participants (the conductor, the teller, the actors, and the audience) and the sequence of events (Fox, 1999). This ritual can easily be monitored in a research framework.

### Life-Crossroads Stories

Life-crossroads is a specific life review technique that uses selected autobiographical memories, self-defining life events, or life periods in which an influential decision and/or a change occurred that had a meaningful effect on the individual’s life course (Keisari and Palgi, 2017). Life-crossroads emphasizes the idea that a life story is built by integrating the meaningful and influential decisions/changes that delineate one’s life events into a life narrative (Bauer and McAdams, 2004). Bringing to life the life-crossroads stories in drama therapy enables an in-depth exploration of the individual’s identity, meaning, and relations with the environment in a concise and condensed manner. In the studies reported here, the participants’ life-crossroads stories were explored mostly via playback theater, as a drama therapy approach that led the therapeutic process.

### General Characteristics of the Four Studies

As shown in Table 1, three studies were based on playback theater as the primary approach to the drama therapy group process (Keisari et al., 2018, 2020a, b) and one was based on a set of drama therapy techniques, including playback theater (Keisari and Palgi, 2017).

Three studies involved participants with normal cognitive functioning in various settings. Few had previous experience with drama or theater. These three studies were based on the exploration of the participants’ life-crossroads stories in dramatic reality (Keisari and Palgi, 2017; Keisari et al., 2020a, b). In one study the participants were therapists who conducted playback theater groups with the aging population (Keisari et al., 2018).

The two quantitative studies directly evaluated the effects of the integration between drama therapy and life-review on different aspects of the participants’ mental health and mental illness (Keisari and Palgi, 2017; Keisari et al., 2020b). The first study used a pre–post study design (Keisari and Palgi, 2017), and the most recent (Keisari et al., 2020b) consolidated the design using a randomized controlled trial with a follow-up examination, a larger sample and a wider range of mental health indices to examine the effect of the intervention.

The two qualitative studies explored the phenomenological characteristics of playback theater as a drama therapy approach for older adults. These two studies were defined to identify the key concepts, and understand the mechanisms that led to transformation (Keisari and Palgi, 2017; Keisari et al., 2020b) and examined the points of view of the conductors, the participants and the staff members. The first qualitative study (Keisari et al., 2018) generated a grounded theoretical model (Strauss and Corbin, 1990) based on the experiences of eight therapists who conduct playback theater with the aging population in several settings. The most recent focused on the integration between playback theater and life-review therapy (Keisari et al., 2020a). This was an art-based study design (Sajnani, 2012; Leavy, 2015), which explored the participants’ experiences by analyzing their relationship to the artistic expression (the dramatic improvisation) and its creative processes. This qualitative study used various data resources to explore the experience of participating in the process including videos that capture the lived experience of the dramatic creative process, semi-structured interviews with the participants and focus groups with staff members.

## Findings

### Positive Effects on Mental Health

Two studies confirmed the positive effect of the integration of life-review and drama therapy for older adults on various indices of mental health. The first (Keisari and Palgi, 2017) reported the effectiveness of a 12-week integrative intervention that combined life-review and drama therapy in improving sense of meaning in life, self-acceptance, successful aging, relationship with others and led to a decrease in depressive symptoms. The second study (Keisari et al., 2020b) indicated that a structured 12-week group intervention that integrates life review with playback theater induced a strong and persistent improvement in self-acceptance, personal growth, relationships with others, satisfaction with relationships, current well-being, positive affect, meaning in life, satisfaction with life, and self-esteem as well as a decline in depressive symptoms. This improvement remained stable 3 months after the intervention. However, the components of negative affect and loneliness were more durable and did not change throughout the experiment. Negative affect and loneliness were high and then decreased, but the follow-up values showed a slight return to baseline levels. The qualitative findings support the claim of transformations that occurred over the course of the process, which led to positive effects on the participants’ mental health (Keisari et al., 2018, 2020a).

### The Evolution of the Life-Story

The qualitative results underscored the transformative potential of the life-story to evolve over the course of the process (Keisari et al., 2018, 2020a). Both studies show how dramatic improvisation brought past memories to life and transformed them into a vivid tangible lived experience that engaged all group members as actors and spectators. The results showed how such an experience provided testimony and acknowledgment of the teller’s life-story. In addition, the results point to the flexibility and spontaneity of the improvised dramatic action as a main component that encouraged the emergence of new meanings and perspectives to co-evolve. The ability to create various perspectives on the story enhanced the participants’ reflective point of view as was positioning the teller as a spectator (Keisari et al., 2018, 2020a).

The results also relate to working with self-defining memories of life-crossroads at the core of the dramatic action. The results showed how positioning the life-crossroads stories in the dramatic improvisation enables the participants to reach and explore their main roles in life, which are essential for self-definition. The results and examples that were presented in three of the studies indicated that working with the main roles in life in the dramatic reality reinforced an integrative view of the self in a more condensed manner. The integrative view was achieved through the dramatic improvisation which makes it possible to hold different kinds of roles from different life-crossroads at the same time (Keisari and Palgi, 2017; Keisari et al., 2020a, b).

The results from both the conductors’ and the participants’ perspectives pointed to the participants’ need to bring life-crossroads stories that represent unfinished conflicts from the past to the group, and the potential of the process to help them come to term with these unresolved conflicts. This is a meaningful developmental task in aging (Butler, 1963; Erikson, 1982). The findings suggested that this process helped the participants to process unresolved conflicts through: (1) the flexibility of the dramatic reality to hold multiple perspectives on the events simultaneously, including the perspective of other characters in the story. This allowed the teller to experience and understand the conflict from the point of view of others, and led to the resolution of conflicts with meaningful others (Keisari et al., 2018); (2) the recreation of the conflictual life-crossroads by others, in a tangible dramatic expression, served to construct a new shared experience for the teller, which was fused to the old memory and reshaped their inner experience in a more positive and acceptable manner (Keisari et al., 2020a).

The protocols presented in three of the studies emphasized the flexibility of the process to enable the tellers to redirect the improvisation of their life-crossroads in a reflective manner by changing roles, switching their positions on stage, and exploring alternative versions of the story, thus influencing their identity cohesion and perceptions of meaning in life (Keisari and Palgi, 2017; Keisari et al., 2020a, b).

The transformative aspects of the process in terms of the evolution of the life-story, as observed primarily through the qualitative results, can also help account for the improvement in mental health indices of *meaning in life*, *self-acceptance*, *relationship with others*, *self-esteem, satisfaction with life*, *well-being*, and *depressive symptoms* in a very short period (12-weeks) (Keisari and Palgi, 2017; Keisari et al., 2020b). Finally, the qualitative results of one study also pointed to the challenges of the process that were mostly related to the emotional difficulties that occurred in processing traumatic stories in a group. Another challenge was its limited duration, which did not allow the participants to bring up additional stories in the group (Keisari et al., 2020a).

### The Evolution of the Improvised Dramatic Expression

The qualitative results were indicative of the evolution of the improvised dramatic expression and its transformative values (Keisari et al., 2018, 2020a). Both studies indicated that engagement in improvised dramatic action generates pleasure, fun, excitement, self-expression, competence, and personal growth. The results also indicated that the embodied expression of the personal story enlists the aging body, a basic requirement for theatrical creation. In addition, while aging is often characterized by the loss of social roles, the results showed that this setting provided the participates with essential roles that involved giving to others, contributing to their own communities, and being emotionally involved in the group. Two of the studies indicated on the participants’ commitment to the process, that was reflected by their consistent presence in the sessions (Keisari et al., 2020a, b).

These transformative values are in line with the values of general drama and theater participation in late life described in the literature (Basting, 1998; Bernard and Rickett, 2017). The studies in this research project support these results by suggesting additional values that provide evidence-based principles for a short and condensed improvised theater participation process that can explore life-stories through dramatic action, without scripts or rehearsals. In addition, the results went beyond the group process by describing how these qualities were incorporated in the daily routine of the community. Participants felt they were able to express themselves more freely in the family and in the community (Keisari et al., 2020a). This experience of competence, joy, self-expression and personal growth can explain the enhancement of mental health indices such as *meaning in life*, *self-acceptance*, *personal growth*, *well-being*, *positive affect*, and *self-esteem* (Keisari and Palgi, 2017; Keisari et al., 2020b). Finally, the qualitative results of one study also pointed to the challenges of the process that were mostly related to the fact that sometimes the theater improvisations were missing aesthetic dramatic qualities (Keisari et al., 2020a).

### The Expansion of Social Engagement

The results of the qualitative studies showed that playing in the life-stories of others creates empathic attunement toward the teller in a way that led to a deep connection between the actor-participant and the teller. The results indicated that this empathic attunement enhances a sense of connectedness in a shared experience that occurred in the dramatic improvisation (Keisari et al., 2018, 2020a). The results also showed that during the creative process, most of the participants could identify their own personal experiences in the teller’s story in a manner that fused the theatrical improvisation with their own life stories to create a unified collective experience: a fusion of experiences (Keisari et al., 2020a). These aspects of the process; namely, the empathic attunement toward the other, the shared experience and the fusion of experiences led to the expansion of the participants’ social engagement. Their social engagement was reflected in the group and later in the adult day center community and family. Given the fact that a positive relationship with others and social engagement are considered to be key factors in health while aging (Cherry et al., 2013; Haslam et al., 2014), this transformative aspect is significant. This was also reflected in the quantitative measures of *satisfaction with relationships* and *relationships with others*, which were significantly improved at the end of the intervention and continued to remain stable 3 months later (Keisari and Palgi, 2017; Keisari et al., 2020b). However, although the positive aspects of social engagement improved, *loneliness* was found to be more persistent over the course of the process. The experiences of loneliness may be more resistant, since the daily lives of many of the adult day center members may have partially impeded them from capitalizing on improvements that occurred as a result of the intervention (such as ongoing coping with the loss of a spouse, the loss of close friends, physical limitations that make social encounters more difficult and others).

## The Multidimensional Model

Taken together, the results from the four studies suggest that the participants underwent significant transformative processes in a relatively short time (Keisari and Palgi, 2017; Keisari et al., 2018, 2020a, b). Therefore, playback theater group participation, integrated with life-review therapy, can be regarded as a drama therapy intervention for the aging population, with solid therapeutic potential. The qualitative findings from two studies–the conductors’ point of view and the participants and staff members’ points of view–complement each other and reflect the main aspects of potential transformations the process induces (Keisari et al., 2018, 2020a). These results are further corroborated by the improvement in mental health indices, revealed in the quantitative studies (Keisari and Palgi, 2017; Keisari et al., 2020b). Furthermore, the improvement in mental health aspects that lasted 3 months after the termination of the process, might indicate that the transformative aspects of the process were internalized, and a re-construction of the subjective experience was indeed created.

**FIGURE 1:**
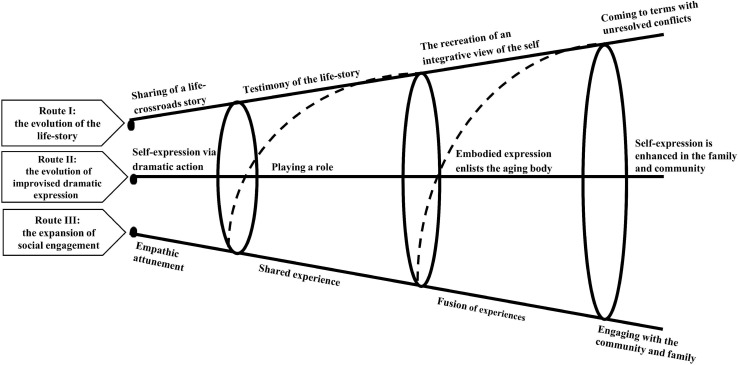
The transformative potential of the life-review drama therapy model, following Corley (2010).

The results of these four studies, when combined, yields a multi-dimensional perspective of the creative therapeutic process, which points to three potential transformative routes that occur over the course of the therapeutic process (Figure 1): (1) the evolution of the life-story that undergoes from the initial stage of sharing it in the group, through its testimonial value, the recreation of an integrative view of the self in the dramatic improvisation, up to the last stage of coming to terms with unresolved conflicts; (2) the evolution of improvised dramatic expression and its therapeutic values from the initial stage self-expression via the dramatic action, which is strengthened in the group through the dramatic roles, the enlisting of the aging body and the self-expression that is reinforced in the community and family; (3) the expansion of social engagement, which starts from the empathic attunement to one another, that develops into a shared experience in the dramatic improvisation, which then evolves to a fusion of experiences, and toward more rewarding social engagement in the community and family.

Each route illustrates the potential transformation the individual might undergo from the initial stage of experience in the group to the more advanced stages of the process where the routes overlap and nourish each other, potentially embracing both body and mind, the self and the other. These three transformative routes in the model were inspired and drawn from Corley’s (2010) “experience, expression and engagement” model, which describes the transformative potential of creativity in late life. Corley’s model (Corley, 2010) refers to the long-term engagement of three aging women who survived the Holocaust with visual art. The adaptation of the model presented here describes the distinctive transformative potential of the integration of life-review and playback theater as a short-term drama therapy intervention while aging.

Each circle in the model depicts the ways in which each route nourishes the others, and the mutual relationships between the routes that enable integrative transformations. In this sense, the reconstruction of the life-stories was made possible through the evolution of the improvised dramatic expression that revealed new meanings and perspectives in the life story, and was also fostered by the safe and creative atmosphere that evolved during the process. Similarly, the dramatic expression evolved in response to the fact that the participants allowed themselves to engage in others’ life stories through their dramatic action in the improvisation. Likewise, the social engagement transformative route evolved as a result of the mutual sharing and re-creation of life-stories in the group, and the ability to play together and take on the dramatic role of the other.

## Discussion

This article presents a new model that combines life-review with drama therapy for the aging population. The analysis was based on the results of four studies that provide multiple-perspectives on the integration of life-review and playback theater as a drama therapy approach in late life (Keisari and Palgi, 2017; Keisari et al., 2018, 2020a, b). The quantitative findings suggest that the integrative process of playback theater and life review can be regarded as a drama therapy intervention with firm therapeutic potential to enhance mental health while aging (Keisari and Palgi, 2017; Keisari et al., 2020b). The qualitative findings (Keisari et al., 2018, 2020a) provide a better understanding of the process, and support the mechanisms that leads to the positive effects on the participants’ mental health. These results are also innovative in documenting the positive effects of the process beyond the group setting in the surrounding environment of the adult day center communities and the participants’ families, and in the improvement in mental health indices at the 3-month follow up.

This produced an integrative model with a multi-dimensional perspective of the creative therapeutic process. Since these distinctive transformative qualities were induced in the context of drama therapy, and playback theater in particular, it is vital to examine the process through the lens of drama therapy *role theory* (Landy, 1993, 2009).

### Exploring the Model Through the Lens of Role Theory

Aging is characterized by the loss of social roles caused by changes in life circumstances, and thus challenges one’s sense of positive identity (Sneed and Whitbourne, 2005). In addition, ageism–the stereotyping of the aging population, along with the discrimination often directed at them (Whitbourne and Hulicka, 1990; Ayalon and Tesch-Römer, 2017)–is also a factor, which leads to curtailment of older adults’ social roles in society and a decline in their sense of relevance and meaning in their community (Dennis and Thomas, 2007; Ayalon and Tesch-Römer, 2017). Hence, although the aging process requires a certain level of personal adaptation, at the same time, one must strive to maintain a consistent sense of self (Whitbourne, 1996; Sneed and Whitbourne, 2003, 2005). This is also reflected in the theoretical and practical principles of narrative theory, which emphasizes two essential factors needed to maintain a positive narrative identity: *stability*, in the form of continuity and *change*, through personal growth (McAdams, 2019).

Accordingly, the creative process in drama therapy groups that integrates life-review makes two contributions. First, it enables the participants to manifest multiple roles which they may have experienced throughout their lifespan, thus maintaining a sense of continuity. At the same time, it enables the exploration and experiencing of new roles during past present and future life events, through the developmental process of aging. These two contributions are analyzed in more detail below.

#### The Expression of Roles in the Life-Story

Life-crossroads stories, as self-defining memories, capture themes and aspects that are essential for self-definition and meaning (Blagov and Singer, 2004; Keisari and Palgi, 2017; van de Goor et al., 2020). Accordingly, the dramatic roles that appear in the dramatic improvisation of one’s life-crossroads as a tangible aspect of the self (Moreno, 1961) bring to life the core aspects of one’s self-identity. The findings and examples from the qualitative findings showed that by reliving the roles taken on during life-crossroads in the therapeutic-dramatic process, the participants could explore deep aspects of the self and their inter-connectedness.

The results suggest how, on the one hand, reliving these roles on the group stage preserved the participants’ sense of continuity and identity. The teller watches from the sidelines, as his/her roles from the past–as “daughter,” “mother,” “producer,” or “caregiver”–receives acknowledgment and validation for his/her own identity. On the other hand, the findings indicated that the process also entails the discovery of new roles in the life-story as new perspectives emerge. For example, in a theatrical improvisation that was created in a response to a life-crossroads story of a Holocaust survivor, the dramatic roles of “survivor” and “escapee” were added to other dramatic roles from the life course that included “family man” and “creator” (Keisari et al., 2020b). In this sense, a new experience was constructed in the group and a more positive and adaptive story was created, leading to identity-transformation (Ben-Ezra et al., 2010; Palgi et al., 2014).

Moreover, expressing these roles together on the group stage creates an integrative view of the self, by uniting roles from the past, present, and future, and roles that represent the contradictions and paradoxes of life. This process enables the participants to integrate these core roles into a coherent view of the self; at the same time, it encourages the acceptance of contradictions and ambivalence. Both of these aspects of the process are considered to be essential in developing a positive identity (McAdams, 1993, 2001; Harel and Keisari, 2021).

The results showed that another significant aspect of the process lies in the ability of the theatrical improvisation to bring to life the dramatic roles of meaningful others in the teller’s life. The life-crossroads story contains significant relationships, which are essential to one’s identity. The results indicated that the ability to enact the dramatic roles of other meaningful characters in one’s life helps the teller form multiple perspectives about significant life-events (Yaniv, 2012, 2014). The capacity to entertain various perspectives helps the individual overcome unresolved conflicts from the past (Keisari et al., 2018; Harel and Keisari, 2021), which is also essential for the development of a positive identity while aging (Erikson, 1982; Pillemer, 2001).

Crucially, the exploration and expression of central themes in life through the enacted dramatic roles is also made possible through the core concept in drama therapy of *aesthetic distancing* which is defined as the balance between identifications and separations in theatrical improvisation (Landy, 1983, 1996; Jones, 1991). This trade off enables the participants to simultaneously be *close* enough to the experience to be able to affectively enact it (affective action), yet *distant* enough to cognitively reflect upon it. The present, lived improvisational piece expresses tangible and vivid significant aspects of the self through its dramatic roles, whereas the participants can choose the ways they relate these aspects to themselves from a safe distance.

#### The Expression of Roles in the Dramatic Creative Process

Individuals usually attend adult day centers after experiencing a major decline in health and physical functioning, after leaving the labor market, the loss of a loved one or as a result of a general decline in psychological and cognitive functioning (Ellen et al., 2017; Ayalon et al., 2018). Many of the activities in the adult day center seem to imply that the participants are passive service consumers rather than active contributors. In this way, they manifest the “passive,” “dependent,” “patient,” and even “disabled” roles. The results here clearly show that the drama therapy group process allowed the participants to express healthy, creative roles such as “creators,” “contributors,” “explorers,” “directors,” “actors,” and “playwrights,” rather than “dependent-related” roles. These roles stimulated a sense of competence, purposefulness and productivity, while enhancing self-expression and pleasure (Sandel and Johnson, 2014). Taking on various dramatic roles also enlists roles of body expression: a body which stands and dance on the group stage in order to be seen, a body which expresses its voice out loud in order to be heard, and a body which is touched by others (Keisari et al., 2018).

The roles of active partners and contributors awaken aspects of the individual’s past self, from time periods during which these roles were more dominant. By expressing these roles–first in the group and then in the adult day center community and in the family–the participants preserve a sense of self continuity. Other roles, especially roles that concern the creative process such as “actors,” “directors,” and “playwrights,” were often first explored by the participants, most of whom had no experience in theater, thereby enabling a sense of new exploration and personal growth (Keisari et al., 2020a).

In addition, each dramatic role that was taken up and played from the life-stories of others enabled the actor-participants to express and explore different aspects of the self through the distancing mechanism, as many of these aspects are hidden, and are not expressed in daily routines. The “child,” the “orphan,” the “leader,” the “mother,” the “guide” are all tangible aspects of the self and are given an expressive-creative space for exploration through the dramatic creative process.

#### The Expression of Roles in the Group and in the Community

The results showed that sharing life-stories in the groups, playing together, and taking on one another’s dramatic roles enhanced the participants’ attunement to one another, their sense of togetherness, belonging and trust, and their mutual support. The findings indicated that participants experienced themselves as relevant and important to others. Such an experience reflects roles and relationships in the community such as “friend,” “neighbor,” and “partner.” Most of these roles are part of the repertoire expressed during the life span, and the ability to intensively express them in the group enhanced both a sense of self-stability and continuity. The results indicate that group cohesion strengthened the participants’ relationships in other activities in the adult day center routine and then spilled over to their families (Keisari et al., 2020a).

Observing the integrative model through the lens of role theory in drama therapy serves to deepen our understanding of the transformative qualities in the therapeutic process. The new model, which is based on the life-crossroads stories, enables individuals to enact and reflect upon their self-defining roles (Keisari and Palgi, 2017; Keisari et al., 2020a) and thus to express and explore their core identity, find meaning in life, and discover new relationships with the environment.

### Limitations

The main limitation of this model relates to the fact that it is based on studies that were adapted to participants with normal cognitive functioning. The integrative model presented here was found to have significant potential in promoting the engagement of the participants in the community (Keisari et al., 2020a, b), but should include all individuals who attend activities at adult day centers, in order to further engage the community (De Medeiros and Basting, 2014; Roe et al., 2016). Other narrative techniques and methods might be used to explore the memories of participants with dementia in a dramatic creative process (Harel, 2016).

In addition, the quantitative studies did not measure the variables over the course of the meetings; for example, through diaries or distributing questionnaires every few meetings. Therefore, it is possible that the end of the group sessions and the farewell meeting might have had an effect on the indices (for example, loneliness might have been affected by the end of the group). Therefore, it is possible that some of the achievements were not captured correctly. In addition, physical condition and functional limitations were not controlled for, although they are known to affect mental health indices (Schieman and Plickert, 2007; Caputo and Simon, 2013).

### Future Research

Future research would benefit from exploring the impact of the model on a range of aging populations, such as participants with dementia or other neurodegenerative conditions. Since participation in theater was found to increase cognitive functioning while aging (Noice et al., 2015), future studies should also examine the effect of the model on cognitive performance. Since the findings indicated a positive effect on the engagement of the participants in the community, and on group cohesiveness, drama therapy groups based on the model should further be explored as a community-based intervention with implications that include the entire community, including the staff and family members as participants. The model can also be explored in other settings such as continuing care retirement communities (Basting, 2018).

Given the fact that negative affect and loneliness were more durable and did not change over the course of the assessment, it would be important to measure the effect over the longer term within a creative process that encourages learning and growth over time (Basting, 2018). It would also be valuable to measure changes in the indices during the process using diaries, and not just at termination.

The model, through its three transformative routes, indicates the healing potential of improvised dramatic expression. Only a handful of papers have related to the healing potential of improvised dramatic play while aging (without rehearsals and planning ahead) (Stevens, 2012; Zeisel et al., 2018). The current results, along with the preliminary studies in the literature, indicate that future research should invest efforts in exploring the effect of improvised dramatic play while aging as a holistic approach that involves and positively influences both body and mind.

The model points to the healing effects of improvised dramatic expression on the aging body. Embodiment is an essential component of selfhood and identity (Eakin, 1999). The transformative routes of the model indicate that the drama therapy process provided a stage to the aging body, which is influenced by changes in physiological functions, subjective and social perceptions toward aging. The participants and conductors indicated that the process evoked and reinforced the body. This experience may enhance a positive body image, which is associated with favorable psychological outcomes while aging. These include reducing psychological distress, and moderating the adverse psychological effects of feeling close to death, and death anxiety (Bodner and Bergman, 2019). Future research should inquire into the body experiences while aging in the realm of the drama therapy.

### Research Contribution and Implications

This article presents a new evidence-based model in drama therapy for the aging population. It is based on the results of four studies that explored the integration of drama therapy and life-review principles, using playback theater and the life-crossroads concept. The uniqueness of this conceptual analysis lies in the involvement of multiple methodological perspectives, which all showed the effectiveness of the process and the transformative routes that lead to the expansion of the dramatic role repertoire while aging.

While the literature on creative art-based interventions in the community has pointed to its strong potential to promote mental health (De Medeiros and Basting, 2014; Fraser et al., 2015; Dunphy et al., 2019), methodologically sound research which can bridge the gap between health care sectors and the field of community-based arts and art therapies is generally lacking (Feniger-Schaal and Orkibi, 2019). The current findings help close this gap by establishing an integrative model to provide effective and readily available interventions for the aging population.

The findings indicate that drama therapy groups also share features with community-based arts interventions since they enhance the participants’ sense of relevance, contribution and engagement in the community (Stickley and Hoare, 2015; Basting, 2018). Inspired by the ideas of Paulo Freire (1970) and Boal’s (2000) *Theater of the Oppressed*, the drama therapy groups in the present model, which is based on the participants’ life-stories and artistic expressions, encouraged individuals to express their voices within the group and later on in their community.

Finally, the key aspects of the model may provide action therapists with a conceptual foundation with which to better understand the transformative effects of drama therapy in late life. The model provides practical directions, guided by two frameworks: the playback theater ritual and life-review therapy principles. These frameworks can be easily followed in the future by therapists who conduct drama-based interventions with the aging population, clinical gerontologists, and narrative therapists by using the life-crossroads concept in their clinical practice.

## Ethics Statement

The studies involving human participants were reviewed and approved by Ethics Committee of the University of Haifa (confirmation number 130/13 and 197/17). The patients/participants provided their written informed consent to participate in this study.

## Author Contributions

SK initiated and designed this conceptual analysis.

## Conflict of Interest

The author declares that the research was conducted in the absence of any commercial or financial relationships that could be construed as a potential conflict of interest.
